# Police liaison and section 136: comparison of two different approaches

**DOI:** 10.1192/pb.bp.115.052977

**Published:** 2017-04

**Authors:** Oliver Jenkins, Stephen Dye, Franklin Obeng-Asare, Nam Nguyen, Nicola Wright

**Affiliations:** 1 Acute Inpatient Services, Norfolk and Suffolk NHS Foundation Trust; 2 University Hospital Lewisham, London

## Abstract

**Aims and method** Two police liaison and section 136 schemes were developed alongside police services at different sites within the same NHS trust. In one, a mental health nurse worked with frontline police attending incidents related to mental health. The other involved nurses providing advice from the police control room. Section 136 detentions were measured over two 6-month periods (6 months apart) before and after practice change. Data analysed included total numbers of section 136 assessments, outcomes following subsequent assessment, and relevant diagnostic and demographic factors. Association of any change in section 136 total numbers and proportion subsequently admitted was investigated in both sites.

**Results** The model involving a nurse alongside frontline police showed significant reduction in section 136 numbers (38%, *P* < 0.01) as well as greater admission rates (*P* = 0.01). The scheme involving support within the police control room did not show any change in section 136 detention but showed a non-significant (*P* = 0.16) decrease in subsequent admission.

**Clinical implications** Mental health nurses working alongside frontline police officers can help improve section 136 numbers and outcomes.

Section 136 of the Mental Health Act 1983 enables an individual police officer to remove any person found in a place to which the public have access appearing to have a mental disorder and to be in immediate need of care or control to a place of safety, such as a hospital, police station or a purpose-built section 136 unit. The section can only be used if the officer believes it is in the interests of that person or necessary for the protection of others. Although section 136 can be an important care pathway to enable an individual to receive appropriate support, it can also be a very distressing experience for them – some report feeling criminalised and punished for having a mental illness.^1^ Its use is also costly given that it requires the input of an approved mental health professional (AMHP) and two doctors for the assessment, plus police and often nursing time to manage the detained person and staff the place of safety.

## Background and aims

The Mental Health Act Commission has highlighted the challenge of collating data on the use of section 136,^2^ but figures have only been gathered at a national level by the Health and Social Care Information Centre over the past 5 years.^3^ These combine police and mental health records on section 136 detentions but have limited detail on outcomes. At a national level the rates of section 136 detentions to places of safety between 2010/2011 and 2014/2015 increased from 14 111 to 19 406.^3^ Attempts to combine inconsistently recorded data have shown a steady upward trend at a rate greater than has been seen with use of the other Mental Health Act sections for detention.^4^

Significant variation is seen regionally in section 136 use. Outcomes from such detentions, often seen as markers of how appropriately the detention has been applied, are also seen to vary from region to region: from areas where around 60% are admitted to hospital^5^ to those where 68% are discharged.^6^ While theories to explain these variations have included a rural/urban divide, police culture, socioeconomic deprivation and diagnostic factors,^7^ the available data have been limited in both accuracy and detail.

In response to concerns about the growing use of this police power and regional variation, there has been a national drive to improve inter-agency working between police and mental health services. Both the Bradley Report^8^ and the Crisis Care Concordat^9^ placed emphasis on local agencies working more closely to improve the experience of individuals in a mental health crisis and to intervene as early as possible within the criminal justice system. Areas have responded differently to the challenge. This paper analyses various aspects of section 136 and changes observed with different ways of inter-agency working within two areas of the same National Health Service (NHS) trust, in Ipswich and Norwich.

The local response in Ipswich has been a Commissioning for Quality and Innovation (CQUIN)-funded pilot scheme developed in partnership between Norfolk and Suffolk NHS Foundation Trust (NSFT) and Suffolk Constabulary. The scheme commenced in April 2014 and involves two experienced mental health nurses working on alternate shifts alongside frontline police officers, 7 days a week from 14:00 until 00:00, to help assess and appropriately divert members of the public who present with potential mental health-related emergencies. Accompanied by a police officer (individuals vary with police rota), they are seen as the initial point of contact for officers attending a potential mental health-related event. They are able to perform face-to-face assessments in a dedicated police car but can also provide advice to other officers over the phone.

Norwich was chosen as the control site as it was closely matched by size, ethnicity and measures of deprivation (Table 1). Norwich also established a police liaison service (funded by the police service) to address the same national concerns. It employed 4 mental health nurses on rotation providing support to the local police force between 8:00 and 22:00, 7 days a week. However, they were based in the police control room and offered telephone advice to police officers without face-to-face contact with the public.

**Table 1:** Results: population parameters^a^

	Ipswich	Norwich
Population	442 000^b^	483 000^c^

Index of deprivation ranking^d^	72	61

White British, %	82.94	83.65

a. 2011 census data.^10^

b. Ipswich, Suffolk Coastal, Babergh and Mid-Suffolk local authorities.

c. Norwich, North Norfolk, South Norfolk and Broadland local authorities.

d. Lower value indicates higher deprivation.

The aims of this study were to examine changes in and between Ipswich and Norwich regarding section 136 detentions and hospital admission rates of detained individuals. This would enable us to build on past research and consider what factors were driving the use of section 136 locally, and in turn better understand any impact the police liaison projects may have had.

## Method

This retrospective study compared numbers and outcomes of section 136 assessments, characteristics of detained individuals and some follow-up data. Information was obtained prior to and following differing changed practices within the trusts for the two areas. Numbers of section 136 detentions were gained from local section 136 suite records and cross-referenced with data gathered at trust level in an attempt to capture all section 136 assessments in Ipswich and Norwich. Further data pertaining to each individual were then collected retrospectively from hospital records using electronic notes (Epex in Ipswich and Carenotes in Norwich). Data were gathered for two 6-month periods: 1 June – 30 November 2013 and 1 June – 30 November 2014. This was to limit any impact on results of the preparation for and introduction of services. This also allowed for comparison of the same 6-month cycle (a year apart) pre- and post-intervention in both areas and between areas.

The project was viewed as service evaluation by the trust's research and development department and thus did not require ethics approval. The exact information gathered and hypotheses to be tested were agreed at the planning stage. Data were entered into Minitab (version 16) to allow for appropriate statistical analysis. The exact data collection questions can be found in Box 1.

Such a study set-up meant that each location had a control group prior to intervention and an experimental group post-intervention. Analysis was conducted pre- *v.* post-intervention in both locations and between the locations. Null hypotheses were that there were no differences between locations or between pre- and post-intervention. Population sizes covered by each section 136 suite were established by combining police force estimates of the locality from which officers were detaining people and the 2011 census local authority population sizes.^10^ Chi-squared statistical tests were used where appropriate. We used *t*-tests when comparing section 136 numbers per 100 000 population, Fisher's exact test for comparing proportions, and the Mantel–Haenszel procedure to identify possible confounding factors.

## Results

### Demographics

As seen in Table 2, there were no significant differences in the study participants' age or gender pre-intervention compared with post-intervention in either area individually or between the areas. There were no significant differences in ethnicity either, with the overwhelming majority of individuals assessed being of White British background.

**Table 2:** Results of section 136 assessments in two study areas^a^

Measure	Ipswich			Norwich			Between-area difference, *P*		
	Pre-	Post-	Within-area difference *P*	Pre-	Post-	Within-area difference *P*	Pre-	Post-	Total
Section 136 assessments,^b^ *n* (%)	169 (77)	104 (47)	**0.01**^c^	87 (36)	93 (39)	0.82^c^	**<0.01**	0.39	**0.01**

Age, years: mean (s.d.)	34.7 (13.3)	37.5 (14.9)	0.12^d^	37.7 (14.0)	37.7 (14.0)	0.98^d^	0.10^d^	0.94^d^	0.15^d^

Males, %	58.0	51.0	0.26	47.6	52.7	0.50	0.12	0.80	0.30

Contact with CMHS in past 2 weeks, *n* (%)	82 (48.5)	52 (50.0)	0.53	52 (65.0)	40 (44.0)	**0.01**	**0.01**	0.40	0.33

Admitted, *n* (%)	40 (23.7)	39 (37.5)	**0.01**	29 (33.3)	22 (23.7)	0.16	0.10	**0.04**	0.89
Admitted under Mental Health Act, *n* (%)	23 (57.5)	23 (59.0)	0.89	16 (55.2)	17 (77.2)	0.10	0.85	0.15	0.46

Assessed during ‘presumed triage hours’, *n* (%)	122 (72.2)	72 (69.2)	0.15^e^	–	–	–	–	–	–
Admitted, *n* (%)	31 (25.4)	28 (38.9)	**<0.01**^e^	–	–	–	–	–	–

Assessed and admitted in ‘non-triage hours’, *n* (%)	9 (19.1)	11 (34.4)	**<0.01**^e^	–	–	–	–	–	–

No mental illness following section 136 assessment, *n* (%)	39 (23.1)	15 (14.4)	0.08	8 (9.2)	25 (26.9)	**<0.01**	**0.01**	**0.03**	**<0.01**

Not admitted
Offered follow-up by secondary MHS, *n* (%)	94 (72.9)	56 (86.2)	**0.04**	42 (72.4)	37 (52.1)	**0.02**	0.95	**<0.01**	**<0.01**
Attended first appointment, *n* (%)	58 (61.7)	50 (89.3)	**<0.01**	30 (71.4)	27 (73.0)	0.88	0.27	**0.04**	0.98
⩾1 further section 136 assessment in subsequent 4 weeks, *n* (%)	27 (20.9)	8 (12.3)	0.14	14 (28.0)	1 (1.4)	**0.01**	0.62	**<0.01**	0.12

CMHS, community mental health services; MHS, mental health services. Bold denotes significance.

a. χ^2^ tests unless indicated otherwise.

b. Per year per 100 000 population.

c. Fisher's exact test for equality of two proportions using figures per 100 000 population per year.

d. Two-sample *t*-test of difference of mean with null hypothesis of no difference.

e. Fisher's exact test for equality of two proportions using the percentage figures.

### Numbers of section 136 assessments

**Fig. 1:**
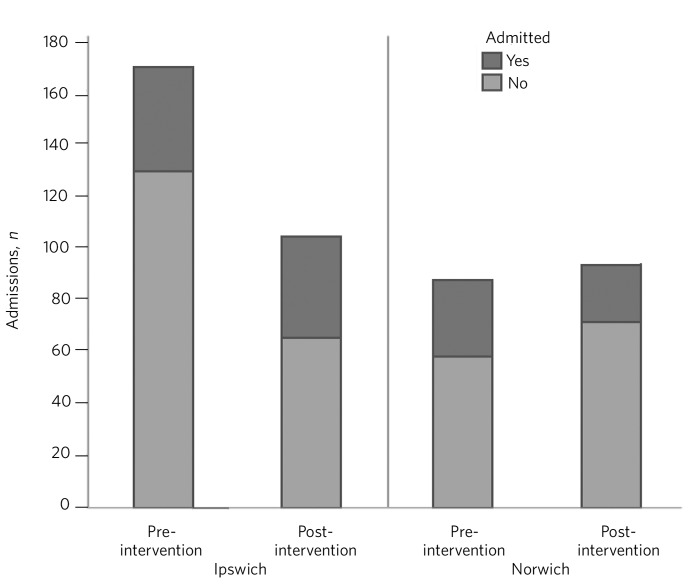
Section 136 assessments and subsequent admissions.

There was strong evidence to suggest, over the total time periods, that Norwich had proportionately fewer section 136 assessments per 100 000 population than Ipswich (*P* = 0.01). This difference was greater in the pre-intervention period. Between the two 6-month periods there was a small, non-significant increase in those detained under section 136 in Norwich but a large reduction in section 136 assessments in Ipswich post-intervention (*P* = 0.01) (Table 2 and Fig. 1).

**Box 1** Survey questions

1. Pre- or post-intervention?
2. Ipswich or Norwich?
3. Date of assessment?
4. Time of assessment?
5. Gender?
6. Age?
7. Ethnicity?
8. Broad ICD-10 category?
9. Number of section 136 assessments the individual had in the previous 6 months?
10. Any contact with mental health services in past 2 weeks?
11. If so, what type of contact?
12. Main reason for section 136?
13. Any specified location?
14. Was the individual admitted?
15. If so, was this under detention?
16. If admitted, what was the length of admission?
17. If not admitted, was follow-up offered by secondary mental health services?
18. If so, was the first appointment complied with?
19. If not admitted, was the individual subject to a further section 136 assessment within the subsequent 4 weeks?

### Admission

Over all the data collected, there was no significant difference in admission to hospital following section 136 assessment between Ipswich and Norwich. Prior to intervention, although admission was less likely in Ipswich than in Norwich, this was non-significant. In Ipswich there was a significant change in admission post-intervention (*P* = 0.01), with a higher conversion to admission. Post-intervention, there was a difference between Ipswich and Norwich (*P* = 0.04), with higher admission in the former. Thus the interventions were associated with a proportionate increase in admissions following section 136 in Ipswich. Although data suggested that the admission proportion decreased in Norwich, this was non-significant (*P* = 0.16).

Apart from weak, non-significant evidence to suggest that in Norwich those admitted post-intervention were more likely to have been detained than pre-admission (*P* = 0.10), there was no other association between detention under the Mental Health Act following admission and pre- and post-intervention status.

### Contact with community mental health services

Any contact with community mental health services (CMHS) in the 2 weeks prior to section 136 assessment was measured. In Norwich, data provided strong evidence that in the pre-intervention period there were more individuals who had some contact with CMHS than in the post-intervention period (*P* = 0.01) and when compared with Ipswich (*P* = 0.01). In Ipswich there was no evidence of any difference between contact pre- *v.* post-intervention.

For those individuals who were not admitted to hospital following section 136 assessment, the proportion that had at least one subsequent 136 assessment in the following 4 weeks decreased in both sites, but the change was significant only in Norwich (*P* < 0.01 *v. P* = 0.14 in Ipswich).

In Ipswich there was moderate evidence to suggest that, if not admitted, people were more likely to be offered follow-up from secondary mental health services post-intervention than pre-intervention (*P* = 0.04). If follow-up was offered in Ipswich, there was strong evidence to suggest that the first follow-up contact was more likely to be kept post-intervention than pre-intervention (*P* < 0.01). In Norwich there was evidence that a person was more likely to be offered follow-up prior to as opposed to after the intervention (*P* = 0.02), but no evidence to suggest any difference between compliance rates pre- *v.* post-intervention.

### Diagnosis

There was weak non-significant evidence to suggest that people assessed in Ipswich were more likely to have been deemed to have ‘no mental illness’ prior to the intervention compared with post-intervention (*P* = 0.08), but in Norwich there was strong evidence to the contrary (*P* < 0.01).

**Fig. 2:**
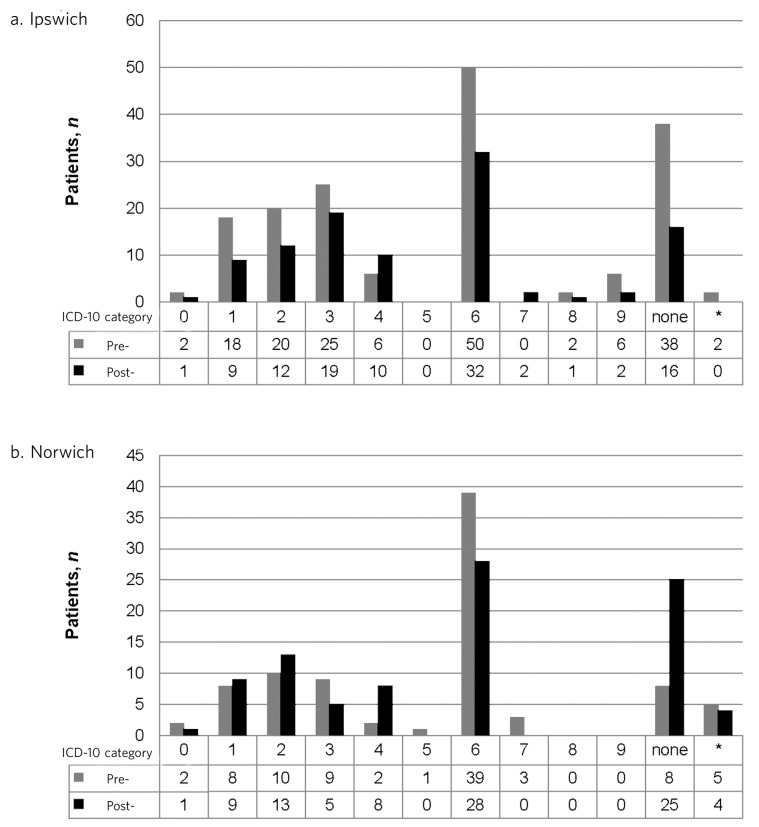
Broad ICD-10 diagnostic categories. a. Not recorded. ICD categories are the first number within the ICD-10 classification: 0 – organic, including symptomatic, mental disorders; 1 – mental and behavioural disorders due to psychoactive substance use; 2 – schizophrenia, schizotypal and delusional disorders; 3 – mood (affective disorders); 4 – neurotic, stress-related and somatoform disorders; 5 – behavioural syndromes associated with physiological disturbances and physical factors; 6 – disorders of adult personality and behaviour; 7 – mental retardation; 8 – disorders of psychological development; 9 – behavioural and emotional disorders with onset usually occurring in childhood and adolescence.

Counts of pre- and post-intervention broad ICD-10 categories of individuals assessed from each site are shown in Fig. 2.

### Time of day

We were unable to obtain the exact time of implementation of the actual section 136 order for individuals. Time between implementation and assessment following the order is influenced by many factors. Trust policy states that this should happen within 3 hours.

In Ipswich the triage service was funded for 10 hours per day (between 14:00 and 00:00). As an estimate within this study, following implementation of the order, individuals who were assessed between 15:00 and 03:00 were deemed to have been assessed in a time period in which the triage service was operating. Within these time periods, the numbers assessed and numbers admitted are shown in Table 2 (numbers admitted following assessment out of these time periods are also shown). Thus, a similar proportion were assessed within the triage hours both pre- and post-intervention (72.2% and 69.2%).

As stated above, admission following section 136 in Ipswich was more likely after the triage service commenced. On further analysis this effect was seen to be confounded by assessment within/outside triage times (Mantel-Haenszel odds ratio 5.44, *P* = 0.02) with a significant association in the triage times (*P* = 0.05). This indicated that the triage service intervention had most association with differing admission rates within the triage times.

Unfortunately, due to a large number of missing assessment time data in Norwich, a similar analysis was not deemed feasible.

## Discussion

This retrospective study evaluated the impact of two recently developed police liaison schemes based in closely matched semi-rural areas within a single mental health NHS trust, with a focus on section 136 rates and outcomes before and after the projects were established.

The pre-intervention findings show that Suffolk Constabulary was detaining significantly higher numbers of people for assessment in the Ipswich area than the police force in Norwich. This difference matches the only data available prior to 2013/2014 on section 136 rates for the two regions, from 2005/2006, in which the Independent Police Complaints Commission (IPCC) analysed regional variation across England and found Suffolk to be a medium-rate user and Norfolk a low-rate user of section 136 detentions to police custody.^11^

Well-matched local population demographics and demographic profiles of those detained would indicate that differences are not linked with variation in race, gender or age. The diagnostic profiles of those detained also show little variation between sites. These observations are significant given that a number of studies have identified common factors pertaining to those detained under section 136, such as Black men being over-represented, and the typical individual tending to be a young male, unemployed, with a psychiatric history and diagnosis of schizophrenia.^12^

As police officers are the sole implementers of S136 detentions, their attitudes and training around mental health can be considered an important variable. Qualitative studies have identified high rates of concern among police officers over inadequate training in relation to mental illness,^13^ and poor understanding of their role in relation to section 136.^14^ Informal feedback from the two police forces involved in this study indicates that mental health training is similar and thus would be an unlikely source of variation.

The IPCC report also observed that low-rate forces used alternative powers such as breach of the peace and that well-known ‘suicide spots’ such as seaside cliffs were observed in police force areas with high rates.^11^ Data we have gathered on both police forces show a similar arrest rate (15 *v.* 16 per 1000 population) but a slightly higher crime rate (49.76 *v.* 43.98 per 1000 population) in Suffolk compared with Norfolk.^15^ However, there are also lower levels of policing per 1000 of population in Suffolk (3.11 *v.* 3.33).^15^ The impact of these slight differences is hard to interpret. Ipswich has a locally well-known ‘suicide spot’ but numbers of detentions relating to its locality were not significant.

Findings from the post-intervention data support the theory that a mental health liaison service to the police can have a significant impact on section 136 rates and also suggest which model is more effective. The Ipswich site showed a 38% reduction in the use of the police power during the post-intervention study period. In that time, there were no other significant changes to police or mental health policy or resourcing locally. This reduction is in contrast to the steady increase in the use of section 136 nationally.^16^ The Norwich site with support based solely in the police control room, by contrast, showed no significant change in overall numbers of section 136 detentions. The data provide some possible explanations for this observed difference between the sites.

It could be speculated that the impact of a liaison service with experienced mental health nurses in Ipswich, where rates were higher 10 years ago^11^ and pre-intervention rates were high in this study, was to enable the local constabulary to achieve a greater level of confidence in dealing with mental health-related crises that is already present in Norwich. The detention outcome data in Ipswich may be seen to lend weight to this idea. Post-intervention we observed a proportionate increase in admission rates, an increase in offer of community support if discharged and a reduction in those deemed to have ‘no mental illness’. These outcome measures can be interpreted as markers of a service better able to identify those with mental health needs and, combined with an increase in engagement, suggest it is better at signposting to appropriate services.

By contrast, the Norwich data post-intervention show a proportionate decrease in admission rates, decrease in follow-up being offered and increase in ‘no mental illness’ assessments despite overall numbers remaining approximately the same. It could be inferred that support based in the police control room is only effective for individuals known to mental health services, whereas members of the public unknown to services need to be assessed face-to-face to provide effective input from a specialist service. Our recording of those who had contact with mental health services in the 2 weeks prior to detention showed that for both areas approximately 50% were either known to or actively open to mental health services, which is lower than estimates from previous research of around 75–84%.^12^ The near equal percentage of those in contact with mental health services prior to detention in Ipswich could further suggest that the face-to-face liaison service is able to affect detention rates for both those known to mental health services and those not known.

The data relating to time of assessments in Ipswich suggested that while the liaison service had a greater impact on conversion to admission rates during their working hours, there was a near-even drop in section 136 rates across all hours. This could indicate that multi-agency working has promoted an ability among the local police force to better identify those who should be detained for further assessment. Lending further weight to this perception is the fact that the service in Ipswich was only in operation 76% of the time due to planned leave and sickness.

A follow-up to the Bradley Report claims that similar schemes are producing positive results, including reduced section 136 rates.^17^ We believe that our study provides an evidence base for these as yet unpublished findings, and lends weight to the value and impact of closer inter-agency working between police and mental health services. The breadth of data and comparison of models available in this study may help to guide the development of future schemes and their refinement.

### Limitations

Within this study we gathered data regarding individuals detained to places of safety under section 136 within the two main urban areas in Norfolk and Suffolk, namely Norwich and Ipswich. There will have been a few occasions where these sites were occupied and people were subsequently taken to other localities. Although we can say that both areas are equally resourced and from experience know these numbers to be small, this should be acknowledged as a weakness. Estimations of section 136 rates per population size in each area must be viewed with caution as the local authority boundaries do not equate to areas that the 136 suites serve, and it was difficult to achieve clarity on this. The study could not define the areas where the section 136 detention was made and this may have been outside the area covered by the triage services, which may have led to an underestimate of the impact of either service.

While this study builds towards a better understanding of the model that is most effective in police liaison work, it lacked a detailed analysis of the work done directly by the mental health nurses and police officers involved. Any future studies should include these data alongside qualitative feedback from relevant professionals and individuals to enable a fuller understanding of the impact of such a service.

## References

[1] Riley G Freeman E Laidlaw J Pugh D ‘A frightening experience’: detainees' and carers' experiences of being detained under Section 136 of the Mental Health Act. Med Sci Law 2011; 51: 164–9. 21905573 10.1258/msl.2011.010074

[2] The Mental Health Act Commission In Place of Fear? The Mental Health Act Commission eleventh Biennial Report 2003–2005. TSO (The Stationery Office), 2006.

[3] Health and Social Care Information Centre Inpatients Formally Detained in Hospitals under the Mental Health Act 1983, and Patients Subject to Supervised Community Treatment: p. 4 HSIC, 2015 (http://www.hscic.gov.uk/catalogue/PUB18803/inp-det-m-h-a-1983-sup-com-eng-14-15-rep.pdf).

[4] Department of Health Review of the Operation of S135 and S136 of the Mental Health Act 1983: pp. 20–68. Department of Health, 2014.

[5] Khurram TS Akbar M Prem M Section 136 assessments in Trafford Borough of Manchester. Clin Govern Int J 2011; 16: 29–4.

[6] Greenberg N Lloyd K O'Brien C McIver S Hessford A Donovan M A prospective survey of Section 136 in rural England (Devon and Cornwall). Med Sci Law 2002; 42: 129–34. 12033467 10.1177/002580240204200203

[7] Borschmann R Gillard S Turner K Chambers M O'Brien A Section 136 of the Mental Health Act: a new literature review. Med Sci Law 2010; 50: 34–9. 20349693 10.1258/msl.2009.009004

[8] Department of Health The Bradley Report: Lord Bradley's Review of People with Mental Health Problems or Learning Disabilities in the Criminal Justice System: pp. 17–158. Central Office of Information, 2009 Available at: https://www.rcpsych.ac.uk/pdf/Bradley%20Report11.pdf (accessed 27 October 2015).

[9] Department of Health and Concordat Signatories Mental Health Crisis Care Concordat: Improving Outcomes for People Experiencing Mental Health Crisis: pp. 6–37. HM Government, 2014 (https://www.gov.uk/government/uploads/system/uploads/attachment_data/file/281242/36353_Mental_Health_Crisis_accessible.pdf).

[10] Office for National Statistics 2011 Census: Aggregate Data (England and Wales). UK Data Service Census Support Downloaded from: http://infuse.ukdataservice.ac.uk This information is licensed under the terms of the Open Government Licence (http://www.nationalarchives.gov.uk/doc/open-government-licence/version/2).

[11] Docking M Grace K Bucke T Police Custody as a ‘Place of Safety’: Examining the Use of Section 136 of the Mental Health Act 1983. Independent Police Complaints Commission, 2008 (https://www.ipcc.gov.uk/sites/default/files/Documents/section_136.pdf).

[12] Borschmann RD Gillard S Turner K Lovell K Goodrich-Purnell N Chambers M Demographic and referral patterns of people detained under Section 136 of the Mental Health Act (1983) in a south London mental health trust from 2005 to 2008. Med Sci Law 2010; 50: 15–8. 20349688 10.1258/msl.2009.009003

[13] Dunn J Fahy T Section 136 and the Police. Psychiatr Bull 1987; 11: 224–5.

[14] Lynch RM Simpson M Higson M Grout P Section 136, The Mental Health Act 1983; levels of knowledge among accident and emergency doctors, senior nurses, and police constables. Emerg Med J 2002; 19: 295–300. 12101134 10.1136/emj.19.4.295 PMC1725901

[15] HM Inspectorate of Constabulary Crime and Policing Comparator. HMIC, 2015 Available at: http://www.justiceinspectorates.gov.uk/hmic/crime-and-policing-comparator/ (accessed 27 October 2015).

[16] Keown P Place of safety orders in England: changes in use and outcome, 1984/5 to 2010/11. Psychiatrist 2013; 37: 89–93.

[17] Durcan G Saunders A Gadsby B Hazard A The Bradley Report Five Years On: An Independent Review of Progress to Date and Priorities for Further Development: pp. 15–16. Centre for Mental Health, 2014 (http://cdn.basw.co.uk/upload/basw_15825-2.pdf).

